# Insight into Kidney Function and Microstructure Through Renal MRI—Review of the Literature

**DOI:** 10.3390/bioengineering13040470

**Published:** 2026-04-17

**Authors:** Marcin Majos, Artur Klepaczko, Ilona Kurnatowska

**Affiliations:** 1 Department of Normal and Clinical Anatomy, Medical University of Lodz, 90-136 Łódź, Poland; 2 Medical Electronics Division, Institute of Electronics, Lodz University of Technology, 90-924 Łódź, Poland; 3 Department of Internal Diseases and Transplant Nephrology, Medical University of Lodz, 90-752 Łódź, Poland

**Keywords:** MRI, magnetic resonance, multi-parametric, chronic kidney disease, CKD, kidney function

## Abstract

Chronic kidney disease (CKD) represents a growing medical, diagnostic and social challenge, and it is estimated to effect 8.5–9.8% of the global population and requires expensive modes of treatment, such as hemodialysis or renal transplants. Currently, a diagnosis of CKD is set based on the level of creatinine in the blood, which is the gold standard of renal function diagnostics. Unfortunately, decrease in GFR is secondary to damage of the kidney parenchyma and indicates that the best time to start more aggressive treatment has already passed. Therefore, several non-invasive methods have been proposed for predicting increased risk of CKD progression; however, in most of the cases kidney biopsy is essential. Currently, the greatest hopes for a method that can confirm CKD are associated with the development of MRI, the most tissue-specific imaging method, and it is already proven to be capable to detect inflammatory and edematous changes, fibrosis, as well as perfusion and oxygenation disturbances. Therefore, in our manuscript we decided to present up-to-date knowledge about kidney MRI from a clinical point of view.

## 1. Introduction

From a clinical point of view, kidney disease can be divided into acute kidney disease (AKD) and chronic kidney disease (CKD). While the definition of AKD is not fully established, it is widely accepted that it entails a reduction in kidney function for less than three months. Usually, the cause of AKD is reversible and does not lead to any complications. It can be caused by glomerulonephritis as a local factor, heart failure, NSAID (nonsteroidal anti-inflammatory drugs) overdose and certain infections, injuries or bleeding taking place throughout the body [1].

The chronic form, CKD, is defined as a state where glomerular filtration (GFR) remains below 60 mL/min/1.73 m^2^ for longer than three months, or when GFR level is above this threshold but is associated with microscopic or macroscopic destruction of renal structure. While CKD can be reversible in specific conditions, its natural evolution is mostly characterized by constant, graduate and inevitable loss of renal function ending in kidney failure. The progression of CKD can be accelerated by episodes of acute kidney injury (AKI), causing further damage to renal morphology [1,2].

Fortunately, a number of treatments exist for the primary diseases known to cause CKD, such as chronic glomerulonephritis, chronic pyelonephritis, hypertension, diabetes, autoimmune and Alport diseases. These have been found to effectively slow or even temporarily stop kidney degradation.

However, CKD still represents a growing medical, diagnostic and social challenge, and it is estimated to effect 8.5–9.8% of the global population [3]. Although this value is significantly lower than the prevalence of cardio-vascular and cancer, CKD therapy is thought to generate much greater costs because CKD patients live for a shorter time in full health and require more expensive modes of treatment, such as hemodialysis or renal transplants [4,5].

Currently, a diagnosis of CKD is set based on the level of creatinine in the blood, which is the absolute standard of renal function diagnostics. This value, along with other data such as sex, age and body weight, is used to calculate the GFR. Unfortunately, as a decrease in GFR is secondary to damage of the kidney parenchyma, a fall indicates that the best time to start more aggressive treatment to maintain kidney function has already passed. Therefore, several methods have been proposed for predicting increased risk of CKD progression; these include urine tests revealing proteinuria and other sediment abnormalities, blood tests for electrolyte levels, histopathological and immunological examinations of biopsy material as well as various, modern diagnostic imaging techniques [1].

Up to now, the role of radiological imaging in the management of CKD remains marginal. In the vast majority of cases, it has been limited to ultrasound (US) examination in M-mode, to determine organ size and morphological features, and in Doppler and spectral-Doppler modes, to evaluate vascular distribution and character of blood flow [6]. The use of other modalities, like computed tomography (CT) and magnetic resonance imaging (MRI), are limited to emergency situations or for diagnosis of widely understood abdominal causes not related to the kidney [1,2,7].

However, while these methods can all provide an insight into the progression of renal function loss at different stages of development, only histopathological examinations have been found to provide a clear picture of the current kidney state. However, due to their invasive nature, they are rarely performed in practice.

**Figure 1:**
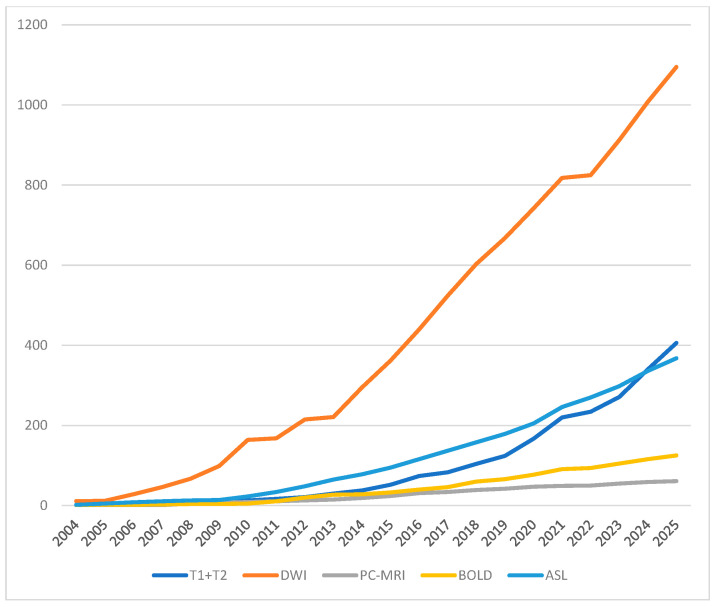
Cumulative number of publication covering subject of renal MRI during PubMed search using strings: renal and MRI and (T1 mapping or T2 mapping or diffusion weighted imaging or phase-contrast MRI or PCMR or blood oxygenation level dependent or arterial spin labeling); the graph covers the last 21 years as before 2004 publications referring to renal MRI are rare.

Currently, the greatest hopes for a method that can confirm CKD are associated with the rapid development of MRI (Figure 1), the most tissue-specific imaging method [8,9]. Therefore, the aim of this article is to present the current state of knowledge regarding the use of advanced MRI techniques for real-time detection of the pathological changes that can occur in the kidneys during CKD progression. These include inflammatory and edematous changes, fibrosis, as well as perfusion and oxygenation disturbances [10,11,12,13]. However, it should be noted that the existing literature on this topic is highly heterogeneous.

This narrative review (to be more precise: “literature review” following cited classification [14]) is based on an online search of the databases PubMed, Cochrane Library, Web of Science and Google Scholar with the time span between 1 of January 1980 to 31 October 2025. The following terms were used in the search: one of the following: (T1 mapping) or (T2 mapping) or (DWI) or (diffusion weighted) or (phase contrast) (PC-MRI) or (phase-contrast MRI) or (phase-contrast magnetic resonance imaging) or (pc magnetic resonance imaging) or (pc-magnetic resonance imaging) or (phase-contrast MR imaging) or (PCMR) or (BOLD) or (blood oxygenation level dependent) or (ASL) or (arterial spin labeling) and (CKD) or (chronic kidney disease). Additionally, we performed manual search through bibliography of included studies for relevant manuscripts. In the final step, we excluded all manuscripts that were not concentrating on renal MRI in chronic kidney disease like focal lesion studies.

## 2. T1 and T2

**Figure 2:**
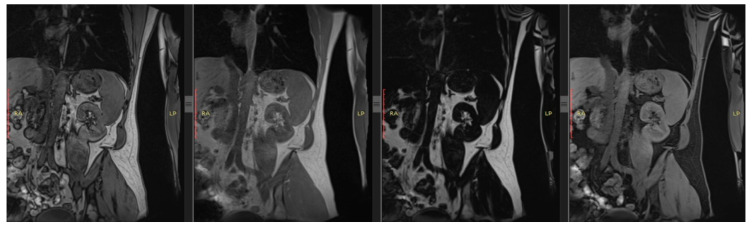
Example of kidney in T1-weighted DIXON image (from the left: in-phase image, opposed-phase image, fat only image, water only image).

**Figure 3:**
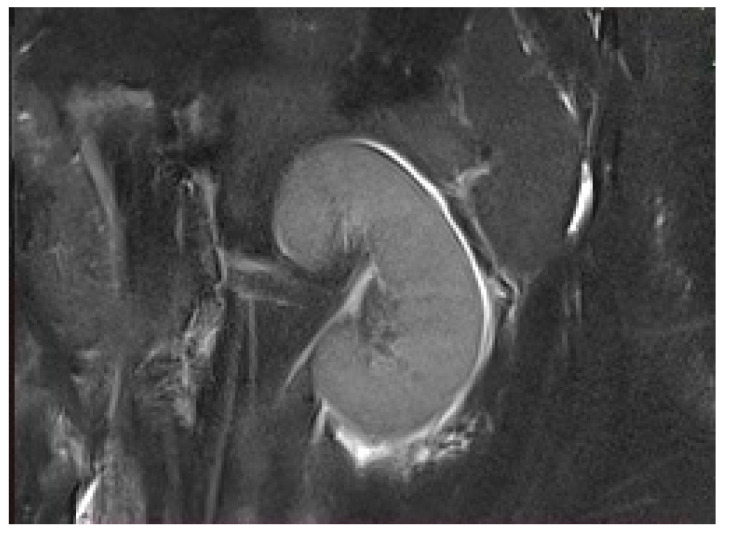
Example of kidney in T2-weighted Haste image.

As early as the 1980s, intensive research began on the clinical utility of T1-weighted images in diagnosing pathologies of both native and transplanted kidneys, with a primary focus on T1 signal intensity changes in the cortex during the development of rejection syndrome [15] (Figure 2 and Figure 3). The results of these initial studies were not convincing; however, more promising findings emerged regarding the effectiveness of T1 in detecting changes in corticomedullary differentiation (CMD), both in the assessment of transplant rejection and in the course of acute tubular necrosis [16,17].

### 2.1. Preclinical Studies

While most studies on the utility of MRI in kidney diagnostics rely on a comparison of both T1- and T2-weighted imaging, such reports have primarily been based on animal studies. Yuasa et al. [18] report prolonged T1 and T2 relaxation times in rabbit kidneys with hypoperfusion caused by renal artery occlusion. These findings were further confirmed in rat models by Pohlmann et al. [19] and mouse models by Hueper K et al. [20]. This is significant because microstructural disturbances in kidney perfusion caused by renal artery stenosis is a key pathomechanism underlying CKD development.

In a mouse model, Franke et al. [21] found that the progression of polycystic kidney disease (PKD) and its treatment can be effectively monitored by T1 and T2 measurements.

Studies have also been conducted to determine the potential of T1- and T2-weighted sequences in evaluating transplanted kidneys, particularly with regard to rejection syndrome. A study on a mouse model [22] found T1 and T2 measurements to be of value in diagnosing of chronic rejection syndrome, while another study on transplanted kidneys in mice, using a multiparametric MRI [23], found greater T1 and T2 signal intensity in the cortex and external medulla to be associated with lymphocytic infiltration and the onset of renal fibrosis.

### 2.2. Clinical Trials

Human based studies analyzing exclusively the T1 or T2 signal remain rare. One study was conducted by Vivian S. Lee et al. [24] on a group of 10 patients found reduced corticomedullary differentiation in individuals with CKD in T1 weighted images to be almost entirely due to changes in cortical signal intensity [24], thus confirming previous studies [15,25,26,27,28]. Studies evaluating T2-weighted imaging in patient populations are rather limited. However, one paper on native kidneys by Tsutomu I [29] identified a significant relationship between hypoxia, degree of kidney fibrosis, T2 signal intensity, and apparent diffusion coefficient (ADC) maps. Also, a particularly interesting study by Mathys et al. [30] demonstrated a correlation between cortical T2 signal intensity and a decline in GFR in kidney transplant patients.

A comparison of MRI findings with histopathological data by Schley et al. [31] found that T1 and T2 relaxation times change as CKD progresses due to hypoperfusion and subsequent fibrosis. However, they concluded that T2-weighted imaging holds greater prognostic value in assessing the progression of chronic kidney disease this suggestion needs more research support.

More recently, the use of MRI technique for evaluating CKD has been significantly improved by the application of texture analysis; briefly, the technique is based on examining the voxel brightness in a given image acquired from a specific sequence. This appears to be an ongoing and significant research trend with substantial clinical potential, and one that may allow objective interpretation of imaging data. In two studies on T1-weighted image textures, the authors demonstrated a correlation between texture parameters and kidney function [32]. The method also allowed healthy individuals to be differentiated from those with CKD secondary to diabetes [33]. Regarding T2-weighted image textures, various algorithms have been designed that can distinguish patient groups depending on the degree of development of CKD, namely Yu Baoting et al. [33], Grzywińska M et al. [34], and Yuki Hara et al. [35]; the methods were also used to effectively determine GFR levels in each subject. The most recent research in this area by Majos et al. [36,37] employed machine learning models based on T1-weighted images and neural network models based on T2-weighted image textures data to successfully detect CKD activity.

## 3. DWI

**Figure 4:**
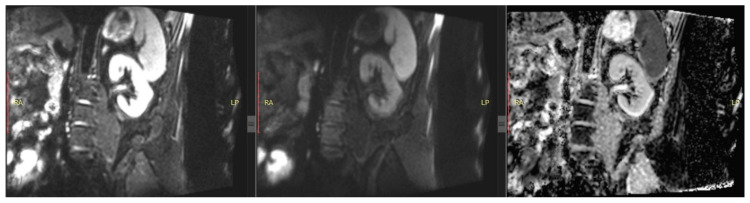
Example of kidney in DWI (b = 0 s/mm^2^, b = 400 s/mm^2^—two images from the left) and ADC map (on the right).

In CKD, the microstructure of the kidneys changes significantly, with a decrease in the number of cells, an increase in the number of fibers and the development of collagen networks. Hence, DWI may represent a powerful approach for evaluating renal microstructure and function (Figure 4).

Studies have found DWI values to effectively distinguish the kidneys of healthy subjects from those with CKD; these have been conducted in diverse groups of patients with different etiologies [38,39,40,41]. Reports also indicate that signal intensity on DWI images correlates with kidney function defined by GFR [42,43,44]. Importantly, several histopathological based studies have found ADC maps to correlate with chronic conditions, including renal fibrosis [45,46]. However, the authors could not clearly interpret the changes observed in DWI or state whether they derive from perfusion disorders or regressive structural changes.

Several interesting studies have been published on CKD associated with the course of diabetes. Various papers have confirmed that it is possible to distinguish diabetic-altered kidneys from healthy ones [47,48], as well as diabetes patients who develop CKD in its course from those developing CKD for other reasons [49]. Equally important, it has been proven that DWI signal intensity can be used to predict the evolution of diabetic CKD as well as the occurrence of macroalbuminuria [47,50].

The evaluation of DWI sequence and ADC maps in the diagnosis of polycystic kidney disease (PKD) still requires further research. Although only few such reports exist, it has been found that the signal associated with kidney parenchyma differs between patients suffering from PKD and healthy controls [51,52], and that DWI images are useful in diagnosing complicated cysts [53].

The value of DWI in assessing the condition of the transplanted kidney appears to be clinically promising. Several studies confirm that DWI analysis can be used to determine the functional status of a transplanted kidney [54] and the development of proteinuria [55].

## 4. Phase Contrast

The PC-MRI sequence can be used to measure renal blood flow (RBF). The data allows indirect assessment of changes in perfusion and microperfusion, which can occur during narrowing of the renal arteries, reduction in the number of nephrons or fibrosis of the intercellular matrix [56].

Renal artery stenosis is known to be a cause of CKD and one of the two most common causes of hypertension; it has however been found to not yield any significant improvement in clinical status [57]. Despite this, attempts have been made to identify special groups of patients [58,59,60], for whom such intervention could be beneficial. So far, this issue remains unresolved.

Several studies have found PC-MRI to be of value in the evaluation of kidney function in CKD. Khatir et al. report that the reduction in perfusion and GFR is not directly proportional and is less than half than the reduction in GFR [61,62,63].

The potential of PC-MRI with regard to polycystic kidney disease has also been evaluated in preliminary studies aimed at confirming the repeatability of results [64,65]. However, two papers have identified a correlation between RBF measured by PC-MRI and kidney function, with one finding it to have predictive value for disease development [64,66].

Only one preliminary report has assessed the value of PC-MRI for evaluating transplanted kidneys [61]; while the estimated RBF values were found to be associated with GFR, this issue requires further research.

## 5. BOLD

**Figure 5:**
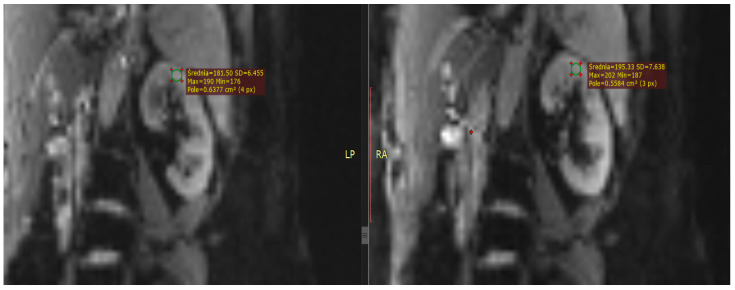
Example of healthy kidney in BOLD maps (on the left peak systolic, on the right peak diastolic).

Although local hypoxia is theorized to be directly related to the development of CKD, the results obtained by studies comparing BOLD data with GFR have not been convincing (Figure 5). Significant data was obtained only when an effective plasma filtration fraction (ERPF) was used [67]. Nevertheless, some studies suggest that BOLD data may be a good biomarker of CKD progression [68,69].

Attempts have been made to evaluate transplanted kidneys using BOLD sequences, which began with the study of Sadowski et al. [70] and by a combination of BOLD data and arterial spin labeling (ASL) [71]. One study found the combination of BOLD data and the use of corticomedullary differentiation factor established on T1- and T2-dependent images to distinguish acute transplant rejection from acute renal tubular necrosis [72,73].

The BOLD sequence can be used to indirectly assess RBF, theoretically making it a suitable tool for assessing changes in the kidney with renal artery stenosis. Several publications have confirmed the correlation between significant stenosis of the renal artery and T2*-weighted signal values [74,75]. Moreover, the BOLD sequence can have a role in monitoring the effects of revascularization [76,77].

## 6. ASL

Numerous studies have evaluated the use of ASL as a single biomarker and as a part of multiparameter studies on chronic kidney disease [78,79,80]. It has demonstrated good accuracy in distinguishing healthy volunteers from patients suffering from CKD. Interestingly, one study on diabetic CKD [81] found ASL to detect early structural changes and to correlate them with kidney function as represented by GFR values; however, it did not indicate that the technique could predict the development of CKD. Despite this, the sequence is sensitive enough to detect changes in kidney blood flow in patients with normal GFR levels [82,83].

Few studies have examined the potential of ASL sequences to evaluate the impact of renal artery stenosis on renal perfusion. The feasibility and repeatability of the technique were verified [84,85] and it was then used as a valuable part of multiparametric research. Hence, ASL appears to have potential diagnostic value both as a single sequence and as a component of multi-factor analyses [86,87,88].

ASL appears to develop in a reliable tool for organ qualification and the evaluation of vessels prior to transplantation [89] as well as for monitoring kidneys after the operation [71,90,91]. A preliminary study by Xue Li et al. [92] found a protocol based on ASL and BOLD sequences to detect renal artery constriction in transplanted kidneys.

Studies on ASL in assessing transplanted kidney function have found estimated RBF to have good correlation with GFR values in the younger population [93] and with long-term kidney transplant recipients [94]. Practically speaking, ASL offers promise when used as part of multiparametric examinations, where it has also been found to demonstrate a good correlations with kidney function [86,87,88].

## 7. Multiparametric MRI

As research on magnetic resonance imaging progressed, researchers have become more interested in the possibility of using diagnostic models containing images of several MRI sequences in diagnostics of CKD.

The natural evolution of CKD determines the great importance of detecting interstitial fibrosis of the kidneys early in its development, and to fulfill this need several studies explored this subject. Friedli et al. observed a correlation between interstitial fibrosis and CDM calculated from T1-weighted and DWI sequences in rat and human models [95]. Meanwhile, Mao et al. proved higher sensitivity in detecting histopathological changes in CKD of an algorithm based on ASL and DWI images than of sole eGFR measurements [96]. Renal MRI was also used in evaluating the specific type of CKD, i.e., in diabetes. In a study concentrated on MR elastography and ASL, Brown et al. proved the ability to assess the development of diabetic nephropathy based on decreasing RBF and decreasing organ stiffness by MRI [97]. In another study, Wang et al. verified the ability to detect changes in the course of diabetes in rat kidney models [98]. Through using data obtained with DTI and Dixon imaging, they were able to effectively detect fat deposits and diffusion disorders characteristic to early stages of diabetic nephropathy.

Never-the-less, it appears that the most widely studied group of CKD patients are kidney transplant recipients. Similarly to patients with native CKD kidneys, the authors evaluated the ability to detect interstitial renal fibrosis in renal grafts. For example, Bane et al., on models based on sequences on DWI, diffusion tensor imaging (DTI), BOLD and T1, and Wang et al., on a model built on DWI and BOLD, proved the ability to detect multiparametric magnetic resonance imaging of organ fibrosis [99,100]. Other authors, such as Bura et al., focused on evaluating kidney function expressed by eGFR values and their correlation with CMD values obtained in T1, T2, and DWI sequences, confirming their strong discriminatory power [101]. In the aforementioned study, the possibility of using CMD in predicting early organ rejection was also evaluated and proven. These findings were confirmed by other reports on the subject, like in a retrospective study conducted by Lui et al. by using a protocol based on DWI and BOLD sequences [102]. Finally, in a study verifying the usefulness of monitoring the function of transplanted kidneys using BOLD and ASL sequences, Niles et al. demonstrated the possibility of using them to assess the effectiveness of losartan treatment [71].

## 8. Conclusions

In summary, all modern MRI sequences can have a role in the diagnosis of kidney diseases and have potential for the evaluation and monitoring of CKD. In addition, all described sequences except PC-MRI seem to be capable to evaluate a transplanted kidneys. T2-dependent, DWI, and ASL sequences are sensitive for CKD caused by diabetes. T2-dependent, DWI and PC-MRI sequences probably will be able to monitor the function of polycystic kidneys and assessing complicated cysts; of these, PC-MRI potentially has predictive value [103].

When examining narrowed renal arteries, all of the listed sequences seem to be sensitive for changes in course and assess items of hemodynamic significance; however, the BOLD and ASL sequences are more likely to be appropriate when qualifying for revascularization and monitoring the effects of treatment [104,105].

## 9. Limitations of the Method

Renal MRI is benefiting patients with chronic kidney disease with its wide range of verified indications and limited contradictions; however, to expand its utility there is great need for multi-center trials concentrated on its predictive value and treatment monitoring. Even though it is not a limitation of a method itself, it is worth mentioning that its accessibility may be not sufficient in less wealthy areas of the world.

Also, MRI manufacturers and diagnostic centers can vary considerably in their choice of acquisition parameters for each of the mentioned sequences; as such, the signal intensity data obtained by specific sequences can also differ between MRI units. Fortunately, there are already some initiatives, like Pulseq [106], trying to overcome this issue. Another valuable initiative trying to overcome this problem is COST action PARENCHYMA [9], which suggests universal protocols of T1 and T2 mapping [107], DWI [108], BOLD [109], phase-contrast [110] and ASL [111].

## Data Availability

No new data were created or analyzed in this study.
